# Mediterranean diet and atrial fibrillation: a case-control study from China

**DOI:** 10.3389/fnut.2024.1433274

**Published:** 2024-10-30

**Authors:** Qian Zhang, Su-Ping Wu, Xu Liu, Yun-Long Wang

**Affiliations:** Department of Cardiology, Beijing Anzhen Hospital, Capital Medical University, Beijing, China

**Keywords:** cardiovascular disease, atrial fibrillation, Mediterranean diet, diet pattern, arrhythmia

## Abstract

**Objective:**

The aim of this study was to assess the association between adherence to Mediterranean diet and the presence of atrial fibrillation (AF) in a Northern Chinese population.

**Methods:**

This study was a single center, case–control study. A total of 952 low risk participants in Beijing Anzhen Hospital from 2016 to 2021 were collected, including 476 patients with first diagnosed of atrial fibrillation and 476 age and sex matched controls. According to the food frequency questionnaire (FFQ), the alternate Mediterranean diet score (AMED) was calculated, which was 0–9 points, indicating the adherence to the Mediterranean diet from low to high.

**Results:**

The average age of the participants was 57.6 ± 9.1 years old, and 70.2% were men. After analyzing every component of AMED, vegetable consumption shows a negative correlation with the risk of AF, whereas alcohol consumption demonstrates a positive correlation with it (OR = 0.61, 95% CI 0.44–0.80, *p* < 0.001; OR = 1.99, 95% CI 1.48–2.58, *p* < 0.001). All patients were grouped according to AMED score. A significant inverse association between AMED and the risk of AF was observed. Compared with participants with AMED<4, the multivariable-adjusted ORs of AF were 0.75 (95% CI 0.55–1.06) for AMED 4–5 and 0.61 (95% CI 0.43–0.89) for AMED ≥6, with a trend in risk (*p* = 0.008). Results were consistent in stratified analyses of gender, age, BMI and smoking.

**Conclusion:**

The Mediterranean diet was inversely associated with the risk of AF in this Northern Chinese population.

## Introduction

Atrial fibrillation (AF), the most prevalent cardiac arrhythmia in adults, exhibits a global incidence ranging from 0.5 to 1%, which increases with age. This disorder precipitates numerous complications, including heart failure, cerebral thromboembolism, systemic emboli, dementia, myocardial infarction, chronic kidney disease, a diminished quality of life, and magnify the overall risk of mortality by twofold (1, 2). Furthermore, the economic impact of AF is substantial and escalating, a survey conducted in the United Kingdom demonstrated that between 1995 and 2000, the proportion of the national healthcare budget attributed to AF-related costs increased from 0.6–1.2% to 0.9–2.4% (3). Despite advancements in pharmacological treatments and catheter-based interventions, the recurrence rate of AF remains alarmingly high.

In recent years, there has been an intensified focus on both preventing the onset of AF and safeguarding against its associated complications (4).

Recent research evidence indicates that changes in lifestyle and diet play a significant role in the prevention and management of AF (5). For instance, contrary to common assumptions, coffee consumption does not correlate with an elevated risk of AF. Indeed, moderate consumption of coffee may confer a protective effect (6, 7). Conversely, alcohol intake is associated with an increased risk of AF, with even moderate consumption (12 g/day) augmenting the incidence of this arrhythmia (8, 9). The consumption of fish or n-3 polyunsaturated fatty acids (PUFAs) does not appear to reduce the risk of AF (10, 11). Plant-based diets, on the other hand, may mitigate numerous risk factors associated with AF (12). However, research explicitly examining the relationship between specific dietary patterns and the incidence of AF remains sparse.

The Mediterranean diet represents a wholesome dietary pattern, distinguished by its copious consumption of plant-based edibles (including fruits, vegetables, legumes, whole grains, and nuts), fish, and olive oil as the principal fat source. The intake of meat and dairy products is comparatively minimal, accompanied by moderate consumption of alcohol. Contemporary research suggests that adherence to the Mediterranean diet correlates with a reduction in overall mortality rates (13), a decreased incidence of cancer (14), cardiovascular and cerebrovascular diseases (15–17), and associated mortality (18). Meta-analyses indicate that each two-point increment in the Mediterranean diet score corresponds to an approximate 10% reduction in the risk of cardiovascular diseases (17). These benefits may stem from the Mediterranean dietary pattern’s ability to reduce atherosclerosis, diminish vascular aging, and enhance endothelial function, among other favorable effects.

Building upon the previous research findings, we postulate an association between the Mediterranean diet and the incidence of AF. We design a case–control study, and aim to provide clinical data for the prevention of AF.

## Methods

### Population

This investigation was a single-center case–control study conducted at Beijing Anzhen Hospital. From 2016 to 2021, it enrolled a cohort of 476 patients who were first diagnosed with AF within 1 month, as classified by the ICD-10. All participants were from northern regions of China, encompassing Beijing, Tianjin, Hebei, Shanxi, Shaanxi, Henan, northern Jiangsu, Shandong, northern Anhui, Heilongjiang, Jilin, and Liaoning. The diagnostic criteria of AF are rhythm record using a standard 12-lead electrocardiogram (ECG) showing AF (absolutely irregular RR intervals and no discernible, repeating P waves). An episode lasts at least 30 s. Or continuous ECG monitoring using skin patch recorders traced of a minimum of 30 s showing heart rhythm of AF (19). Patients presenting with malignant tumors, anemia, thyroid dysfunction, or other cardiac conditions were excluded from the study. Given the established link between cardiovascular diseases (CVD) and metabolic risk factors such as hypertension, diabetes mellitus, and hyperlipidemia (20), we anticipated that the study population would exhibit a lower burden of cardiovascular risk factors. This selection criterion was intended to more accurately assess the isolated impact of dietary patterns on the risk of AF, thereby excluding individuals with hypertension, diabetes mellitus, and hyperlipidemia.

Hypertension was defined as a systolic blood pressure ≥140 mmHg and/or a diastolic blood pressure ≥90 mmHg, or the current use of antihypertensive medications. Hyperlipidemia was characterized by a total cholesterol level ≥240 mg/dL and/or a low-density lipoprotein cholesterol level ≥160 mg/dL, or the current use of cholesterol-lowering medications. Diabetes was identified by a fasting blood sugar level ≥126 mg/dL and/or a non-fasting blood sugar level ≥200 mg/dL, or the current use of antidiabetic medications. The diagnostic criteria for anemia were specified as follows: for adult men, a hemoglobin concentration <120 g/L; for adult women, a hemoglobin concentration <110 g/L, or the ongoing administration of medication for anemia. The diagnosis of thyroid dysfunction was established based on thyroid hormone levels measured upon admission and corroborated by an endocrinologist’s evaluation. Cardiac diseases encompassed congenital heart disease, coronary atherosclerotic heart disease, rheumatic heart disease, heart valvular disease, cardiomyopathy, heart failure, and cardiac arrhythmia. The presence of malignant tumors was determined based on the patient’s medical history.

**Figure 1:**
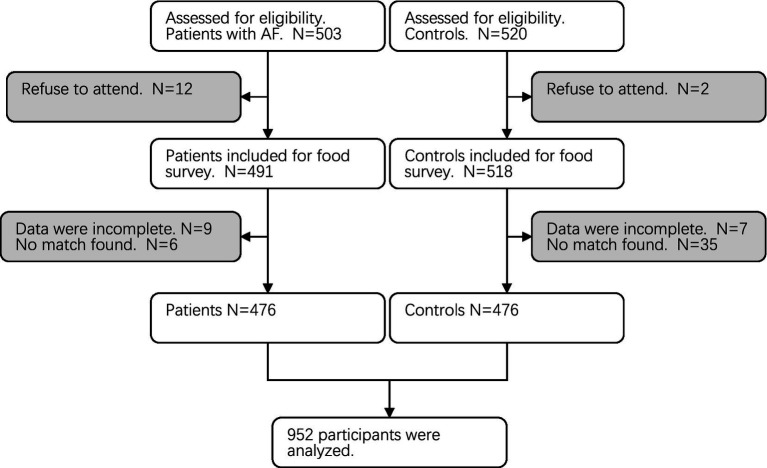
Study flowchart.

The control group consisted of 476 individuals sourced from the Medical Examination Center at Beijing Anzhen Hospital, all originating from northern China. These participants were matched with their case counterparts based on age (±1 year), sex, and household type defined by their residential area (rural or urban) over the past 5 years. The inclusion criteria for control patients stipulated that they must have undergone an electrocardiogram (ECG) within the preceding month, with the results showing no significant abnormalities. To maintain the integrity of the study, individuals in the control group with any history of heart diseases, hypertension, diabetes, hyperlipidemia, malignant tumors, anemia, or thyroid dysfunction were excluded (Figure 1).

### Study design

The selected patients had their dietary patterns, eating habits, smoking status, alcohol consumption, and physical activity levels meticulously collected by investigators who were uniformly trained to ensure consistency in data gathering. Additional medical history and pertinent medical details were extracted from hospitalization or outpatient records, encompassing metrics such as height, weight, blood pressure, heart rate, hemoglobin levels, blood glucose, plasma cholesterol, among others.

Baseline dietary information was obtained using a Food Frequency Questionnaire (FFQ) (21), a validated and semi-quantitative tool designed to capture the types of food consumed and the average frequency of intake on a daily, weekly, and monthly basis. To enhance the accuracy of dietary assessments, patients were occasionally requested to provide photographs of their meals, which aided in the evaluation of portion sizes. Daily nutrient intake was estimated with reference to the China Food Composition Table (Standard Edition). Portion sizes for specific food items were standardized according to the guidelines set forth in the 2016 Chinese Dietary Guidelines (22). Physical activity levels of patients were comprehensively assessed by documenting occupational, commuting, leisure-time, and household activities. The total duration of physical activity was calculated and subsequently converted into metabolic equivalents, providing a quantifiable measure of energy expenditure associated with different forms of physical activity (23).

The Mediterranean Diet Score was meticulously computed for all participants. The Alternate Mediterranean Diet (AMED) score is specifically designed to gauge the adherence of non-Mediterranean populations to the Mediterranean dietary pattern, representing an adaptation of the traditional Mediterranean diet (24). Our analysis concentrated on nine principal foods or nutrient components: vegetables, fruits, legumes, whole grains, nuts, fish, the ratio of monounsaturated fatty acids to saturated fatty acids (MUFA:SFA ratio), red or processed meats, and alcohol. Given controversial relationship between alcohol consumption and AF, alcohol intake for all participants was standardized into alcohol units. In this study, participants reported their alcohol consumption, and a scoring system was employed where individuals received 1 point for alcohol intake below the median for their gender, and 0 points for intake above the median. For the purposes of this analysis, “drinking” was defined as having an alcohol consumption above the gender-specific median. Participants were stratified into three distinct groups based on their AMED scores: those with an AMED score less than 4 (AMED <4 group), those with a score between 4 and 5 (AMED 4–5 group), and those with a score of 6 or higher (AMED ≥6 group). Employing the AMED <4 group as the reference category, we analyzed the relative risk of AF occurrence across the different groups, aiming to elucidate the protective or detrimental effects of adherence to the Mediterranean dietary pattern on the incidence of AF.

### Statistical analysis

The principal objective of this investigation was to evaluate the association between the AMED score and the risk of AF. For the purposes of analysis, the IBM SPSS 25.0 software suite was employed. Continuous variables were delineated as means ± standard deviations or medians, depending on their distribution, and were analyzed using independent t-tests or analysis of variance as appropriate. Categorical variables were reported as frequencies, and their associations were assessed using the chi-square test. To further elucidate the relationship between dietary adherence and AF, significant variables distinguished between the two groups, along with established traditional cardiovascular risk factors, were incorporated into a multivariable logistic regression model. Using the group with an AMED score less than 4 (AMED <4) as the reference category, the odds ratios (ORs) and 95% confidence intervals (CIs) for the AMED 4–5 and AMED ≥6 groups were calculated and analyzed. A *p*-value of less than 0.05 was deemed to indicate statistical significance, underscoring the robustness of the observed correlations.

## Results

### Baseline characteristics

This study included a total of 952 participants (668 males, mean age 57.6 ± 9.1 years). Cases were 476 patients with AF, and controls were 476 patients matched by gender, age, and residential area. Comparing clinical indicators between the two groups, patients in the AF group had higher BMI values (25.3 ± 3.7 vs. 24.5 ± 3.4 kg/m^2^, *p* = 0.016). In terms of lifestyle factors, patients in the AF group had lower AMED scores (4.3 ± 1.4 vs. 4.7 ± 1.5, *p* = 0.001), with no significant statistical differences observed in other variables between the two groups (Table 1).

**Table 1:** Baseline information of participants in AF group and control group.

Characteristic	AF (476) Mean ± SD or *n* (%)	Control (476) Mean ± SD or *n* (%)	*P*
Age (year)	57.6 ± 9.1	57.6 ± 9.1	0.985
Male	334(70.2)	334(70.2)	1.000
Education ≥ high school graduate	310(65.1)	322(67.4)	0.410
Household income ≥ $15,000/year	328(68.9)	349(73.3)	0.133
Urban resident*	383(80.5)	383(80.5)	1.000
Systolic blood pressure (mmHg)	128.2 ± 16.7	126.3 ± 15.8	0.285
Diastolic blood pressure (mmHg)	70.1 ± 12.3	71.4 ± 12.5	0.352
Body mass index (kg/m^2^)	25.3 ± 3.7	24.5 ± 3.4	0.016
AMED score	4.3 ± 1.4	4.7 ± 1.5	0.001
No diet change in past 1 year	453(95.2)	449(94.3)	0.610
Current smoking	120(25.2)	109(22.9)	0.404
Former smoking	22(4.6)	20(4.2)	0.875
Physical activity (MET-h/day)	24.9 ± 16.4	25.3 ± 16.6	0.073

*Residents living in cities in the past 5 years.

### Food group differences

**Figure 2:**
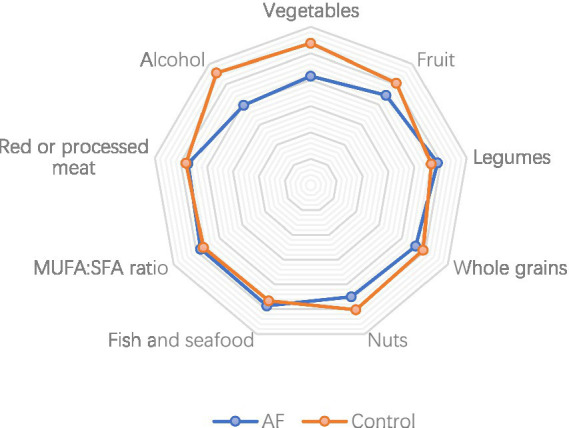
Average scores achieved for items in AMED of AF patients and controls.

Scoring each food type in all patients, analyzing their correlation with AF through uni- and multivariable methods. The risk of AF was inversely associated with the consumption of vegetables (OR = 0.61, 95%CI 0.44–0.80, *p* < 0.001). A risk reduction of borderline significance was discovered for a high consumption of fruits. The risk of AF has a positive correlation with alcohol consumption (OR = 1.99, 95%CI 1.48–2.58, *p* < 0.001). No association was detected for the other dietary items under consideration (Table 2 and Figure 2).

**Table 2:** Odds ratios of atrial fibrillation for every component of the altered Mediterranean diet score.

Components	OR (95% CI)*	*P*	OR (95% CI)**	*P*
Vegetables	0.59(0.46-0.77)	<0.001	0.61(0.44–0.80)	<0.001
Fruit	0.77(0.60–1.00)	0.050	0.78(0.59–1.01)	0.082
Legumes	1.11(0.86–1.42)	0.437	1.10(0.84–1.43)	0.496
Whole grains	0.87(0.68–1.13)	0.300	0.91(0.62–1.18)	0.560
Nuts	0.80(0.62–1.04)	0.092	0.82(0.59–1.07)	0.135
Fish and seafood	1.09(0.84–1.40)	0.517	1.10(0.82–1.41)	0.632
MUFA:SFA ratio	1.05(0.82–1.36)	0.697	1.04(0.80–1.37)	0.773
Red or processed meat	0.97(0.75–1.25)	0.795	0.98(0.74–1.27)	0.814
Alcohol	1.97(1.52–2.55)	<0.001	1.99(1.48–2.58)	<0.001

MUFA, monounsaturated fatty acids; SFA, saturated fatty acid. *Adjusted for sex and age. **Adjusted for sex, age, education, income, residential area, heart rate, blood pressure, BMI, smoking, BMI, diet change in the past year, smoking, physical activity. OR, odds ratio; CI, confidence interval.

### Correlation analysis between AMED and AF

Univariate analysis reveals a negative correlation between AMED and the risk of AF. Decreasing odds ratios (ORs) were discovered for increasing AMED scores, with a significant trend in risk (*p* = 0.005). In the multivariable analysis, this trend persists (*p* = 0.008). Compared with AMED <4, the OR from the fully adjusted model was 0.75 (95%CI 0.55–1.06, *p* = 0.070) for individuals with AMED 4–5 and 0.61 (95%CI 0.43–0.89, *p* = 0.010) for individuals with AMED ≥6 (Table 3).

**Table 3:** Odds ratios of atrial fibrillation by altered Mediterranean diet score group.

Variates	<4	4–5	≥6	*P* for trend
AMED	3.3 ± 1.4	4.3 ± 1.4	6.5 ± 1.5	
Case group	179	196	101	
Control group	142	205	129	
OR (95% CI)*	1.00 (−)	0.76 (0.57–1.02)	0.62 (0.44–0.87)	0.005
OR (95% CI)**	1.00 (−)	0.75 (0.55–1.06)	0.61 (0.43–0.89)	0.008

*Adjusted for sex and age. **Adjusted for sex, age, education, income, residential area, heart rate, blood pressure, BMI, diet change in the past year, smoking, physical activity. OR, odds ratio; CI, confidence interval.

### Stratified analysis

Stratified by gender, age, body mass index, smoking status, a multivariable regression analysis assesses the correlation between AMED and the risk of AF. There is no significant difference in all characteristics after stratification (Table 4).

**Table 4:** Odds ratios of atrial fibrillation for AMED scores in strata of selected covariates.

AMED	<4 OR (95% CI)	4–5 OR (95% CI)	≥6 OR (95% CI)	*P*-trend	*P*-inter-action
Gender					
Men	1.00(−)	0.71(0.50–1.00)	0.57(0.38–0.86)	0.006	0.225
Women	1.00(−)	0.91(0.53–1.57)	0.76(0.41–1.41)	0.385	
Age (years)					
≥60	1.00(−)	0.84(0.52–1.37)	0.83(0.47–1.45)	0.476	0.132
<60	1.00(−)	0.72(0.50–1.36)	0.53(0.34–0.81)	0.003	
Body Mass Index					
≥25	1.00(−)	0.75(0.45–1.25)	0.54(0.30–0.99)	0.045	0.675
<25	1.00(−)	0.77(0.53–1.11)	0.68(0.45–1.04)	0.068	
Smoking					
Yes	1.00(−)	0.68(0.37–1.23)	0.56(0.28–1.14)	0.097	0.538
No	1.00(−)	0.79(0.56–1.10)	0.64(0.43–0.95)	0.024	

OR, odds ratio; CI, confidence interval.

## Discussion

This study demonstrates that adhering to a Mediterranean diet is inversely associated with the risk of AF. Even after controlling for known influencing factors, this correlation persists. Particularly, individuals scoring 6–9 on the AMED scale exhibit a significantly lower risk of AF compared to those scoring 0–3. The origins of the hypothesis that the Mediterranean diet promotes health trace back to observations made in the 1960s of the notably healthier inhabitants of Crete and southern Italy. The traditional Mediterranean diet is characterized by a low content of saturated fatty acids (SFAs) and high levels of monounsaturated fatty acids (MUFAs), carbohydrates, and fiber, derived predominantly from vegetables, fruits, grains, and notably, olive oil. The diet is rich in essential nutrients such as beta-carotene, vitamins C and E, folate, flavonoids, polyphenols, as well as various essential minerals and trace elements, all of which have demonstrated benefits for human health, particularly in the context of cardiovascular diseases (17, 25). Empirical evidence supporting the cardiovascular benefits of the Mediterranean diet includes a longitudinal study with a follow-up period of 4.7 years, which revealed that individuals adhering to a Mediterranean diet supplemented with olive oil exhibited a significantly lower risk of AF compared to those following a low-fat diet (Hazard Ratio = 0.62, 95% Confidence Interval: 0.45–0.85) (26). Further research by Felix suggested, through a small-sample study, that the Mediterranean diet contributes to a reduction in AF risk (27). Moreover, a case–control study involving 800 participants found that the Mediterranean diet score was significantly lower among AF patients compared to controls (22.3 ± 3.1 vs. 27.9 ± 5.6, *p* < 0.001), and those with higher scores were more likely to revert from AF to sinus rhythm (28). Collectively, these findings advocate for the Mediterranean diet as a preventive strategy against cardiac arrhythmias, particularly AF, highlighting its potential as a significant dietary intervention in cardiovascular health management.

Within the nine dietary categories of the Mediterranean diet analyzed in this study, the group diagnosed with AF displayed a significantly elevated alcohol consumption score compared to the control group. The link between alcohol consumption and the incidence of AF is well-documented, chronic heavy alcohol intake markedly heightens the risk of AF, even in the absence of structural heart changes (29, 30). Some observational studies have indicated that consuming two or more alcoholic drinks per day increases the risk of AF by 30% (29). Bazal et al. (30) suggested that light alcohol consumption within the Mediterranean diet pattern does not increase the risk of AF, while Dora et al. (8) argued that even light alcohol consumption slightly elevates the risk. The risk notably increases when alcohol consumption surpasses one drink per day. The exact mechanism by which alcohol triggers AF remains unclear (31, 32), but potential mechanisms include shortening of the atrial refractory period, increased sympathetic nerve excitability, reduced vagal nerve excitability, and alterations in atrial current density (33, 34). Another possible mechanism is that alcohol consumption affects cytoplasmic sulfotransferase activity and histamine levels, Moreover, it raises blood pressure, resulting in atrial remodeling and exacerbating the risk of atrial fibrillation (35).

Increasing evidence supports the close relationship between inflammation and AF, which is associated with the occurrence and maintenance of persistent and paroxysmal AF (36, 37). Possible mechanisms of inflammation-induced AF include oxidative stress, alterations in calcium ion homeostasis, cardiomyocyte apoptosis, and ultimately myocardial fibrosis. Inflammatory levels in the circulatory system or within the heart can predict the onset and recurrence of AF, and regulating inflammatory levels can improve atrial electrophysiological function (38). For example, post-cardiac surgery, an increase in total leukocyte count independently predicts the occurrence of AF, and the application of antioxidant and anti-inflammatory therapies after cardiac surgery can reduce the incidence of AF (39). Plant-based foods (such as fresh vegetables, fruits, etc.) contain anti-inflammatory and antioxidant components that significantly reduce systemic inflammatory responses. Many studies have shown that a plant-based diet can lower levels of plasma C-reactive protein (CRP) (40, 41). Franco found that vegetarians have significantly lower plasma CRP levels compared to omnivores (1.1 mg/L vs. 0.5 mg/L, *p* < 0.05) (42). Some researchers believe that the Mediterranean diet, which involves consuming foods rich in omega-3 fatty acid esters, may help reduce inflammation and improve cardiac function (27).

The occurrence and persistence of AF significantly impact people’s quality of life and mental health. The beneficial effects of the Mediterranean diet on chronic diseases are generally attributed to the rich variety of food intake and the interactions between different food types (43, 44), making it difficult to isolate the effects of individual food types. The Mediterranean diet pattern is believed to improve various metabolic disorders, thereby substantially reducing known risk factors promoting AF. A study in 2014 further supported that the Mediterranean diet has significant cardiovascular benefits. The subjects of this research were elderly atrial fibrillation patients with atherosclerosis. The results showed that a high degree of adherence to the Mediterranean diet was related to reduced platelet activation and decreased production of thromboxane A2 (45). PREDIMED study shows that the Mediterranean diet can reduce cardiovascular events by 30%. Participants have improvements in blood pressure, blood sugar, and blood lipids (28). Given the complexity of human diet, considering diet as a whole and finding a suitable dietary pattern may be more beneficial for the prevention of AF.

This investigation acknowledges certain limitations that warrant consideration. Primarily, the study cohort comprised individuals who were first diagnosed with AF and presented at Anzhen Hospital. Given the extensive geographic expanse and populous nature of the northern region of China, the AF samples may not fully represent the entire demographic of the area. Additionally, potential biases may arise from participants’ recall of their past dietary and lifestyle habits, which could affect the accuracy of the data collected. It is necessary to underscore that this study elucidates a correlation between AMED and AF, but it does not establish a definitive causal relationship. Nevertheless, as society progresses, the medical burden of AF is growing ever more onerous. This study probed into a simple and economical preventive measure and offered notions for subsequent prospective interventional studies.

## Data Availability

The original contributions presented in the study are included in the article/supplementary material, further inquiries can be directed to the corresponding author.
